# Investigating Associations between HLA-DR Genotype, *H. pylori* Infection, and Anti-CagA IgA Seropositivity in a Turkish Gastritis Cohort

**DOI:** 10.3390/genes15030339

**Published:** 2024-03-06

**Authors:** Lokman Karataş, Zeynep Tatar, Eddie A. James, Mukaddes Colakogullari

**Affiliations:** 1 Health Sciences Institution, Istanbul Medipol University, Istanbul 34815, Turkey; lokmankaratas@gmail.com; 2 HLA Laboratory, Istinye University, Istanbul 34010, Turkey; 3 Patomer Pathology Laboratory, Fatih, Istanbul 34096, Turkey; drzeynep1971@gmail.com; 4 Translational Research Program, Benaroya Research Institute, Seattle, WA 98101, USA; 5 Clinical Biochemistry Department, Faculty of Medicine, Izmir Democracy University, Izmir 35140, Turkey

**Keywords:** anti-CagA IgA, *Helicobacter pylori*, HLA DRB1*11:04, HLA DRB1*03:01

## Abstract

*Helicobacter pylori* (*H. pylori*) is associated with gastric inflammation and mucosal antibodies against its cytotoxin-associated gene A (CagA) are protective. Vaccine-elicited immunity against *H. pylori* requires MHC class II expression, indicating that CD4+ T cells are protective. We hypothesized that the HLA-DR genotypes in human populations include protective alleles that more effectively bind immunogenic CagA peptide fragments and susceptible alleles with an impaired capacity to present CagA peptides. We recruited patients (n = 170) admitted for gastroendoscopy procedures and performed high-resolution HLA-DRB1 typing. Serum anti-CagA IgA levels were analyzed by ELISA (23.2% positive) and *H. pylori* classified as positive or negative in gastric mucosal tissue slides (72.9% positive). Pearson Chi-square analysis revealed that *H. pylori* infection was significantly increased in DRB1*11:04-positive individuals (*p* = 0.027). Anti-CagA IgA was significantly decreased in DRB1*11:04 positive individuals (*p* = 0.041). In contrast, anti-CagA IgA was significantly increased in DRB1*03:01 positive individuals (*p* = 0.030). For these HLA-DRB1 alleles of interest, we utilized two in silico prediction methods to compare their capacity to present CagA peptides. Both methods predicted increased numbers of peptides for DRB1*03:01 than DRB1*11:04. In addition, both alleles preferred distinctively different CagA 15mer peptide sequences for high affinity binding. These observations suggest that DRB1*11:04 is a susceptible genotype with impaired CagA immunity, whereas DRB1*03:01 is a protective genotype that promotes enhanced CagA immunity.

## 1. Introduction

*Helicobacter pylori* (*H. pylori*) is a Gram-negative bacterium that, through specific adaptation to inhabit the acidic environment of the stomach, is estimated to infect approximately half of the human population [1]. Despite this wide prevalence, *H. pylori* is not a commensal. Rather, *H. pylori* is classified as an amphibian bacterium with both pathogenic and beneficial effects [2]. The infection has been associated with protection from esophageal eosinophilia, allergy, and inflammatory bowel disease [3,4,5] but associated with susceptibility to peptic ulcers and gastric inflammation [6]. *H. pylori* strains are broadly categorized as cytotoxin-associated gene A (CagA)-positive or -negative. The CagA gene, which is located on a pathogenicity island that encodes disease-associated virulence factors [7], was shown to promote the neoplastic transformation of gastric epithelial cells through tyrosine phosphorylation-dependent and phosphorylation-independent mechanisms [1]. Consequently, CagA-positive *H. pylori* is classified as a group I carcinogen [8]. Furthermore, epidemiologic studies [9,10], prospective cohort studies [11,12], and rodent infection models [13,14] implicate *H. pylori* infection as a major risk factor for the development of gastric cancer.

*H. pylori* lives on the surface of the gastric mucosa and elicits measurable levels of serum antibodies. In particular, immunoglobulin G (IgG) and immunoglobulin A (IgA) against whole-cell or CagA measured in serum or gastric juices were observed at significantly higher levels in *H. pylori*-positive subjects [15]. Comparing different *H. pylori* strains, CagA+ strains have been associated with higher grades of gastric inflammation and an increased risk of peptic ulceration [16]. Multiple lines of evidence suggest that the formation of mucosal antibodies against *H. pylori* infection may be protective. Patients with severe active inflammation had decreased levels of IgA and levels of the inflammatory cytokine IL-8 and IgA were inversely correlated [6], suggesting that IgA protects from *H. pylori*-mediated inflammation and damage. Although CagA-positive *H. pylori* strains are associated peptic ulcers, high titers of anti-CagA antibody were observed in individuals with normal mucosa [17], indicating that these antibodies are not merely indicative of infection and inflammation. In contrast to the negative association seen for CagA, titers of heat shock protein and 25-kDa protein specific IgA in serum and gastric juice were correlated with the histologic grade of gastritis [18]. Vaccination studies in MHC class I and MHC class II deficient mice have demonstrated that protection against *H. pylori* infection was impaired in MHC class II deficient animals [19,20], supporting the relevance of CD4+ T cell responses in protection. The production of IgA by B lymphocytes is most likely dependent on T cell help. To elicit CD4+ T cell responses, *H. pylori*-derived proteins must be taken up by antigen-presenting cells, processed, and presented to T cells. Notably, the MHC locus is the most polymorphic region of the human genome [21]. These polymorphisms are closely grouped within the peptide binding cleft, thereby conferring allele-specific features, such that each HLA protein binds and presents a distinct array of peptides that is complementary to the size, shape, and charge of its binding pockets [22]. The limited number of studies that have investigated CD4+ T cell responses have exclusively identified HLA-DR restricted responses, with no overlap in epitopes between the different alleles studied [23,24,25,26,27]. Therefore, it is plausible that diverse HLA-DR haplotypes present distinct *H. pylori* peptides, with some more effectively presenting immunogenic peptides to CD4+ T cells and eliciting help for antibody formation than others.

In this study, we investigated the relationship between the presence of anti-CagA IgA levels in gastritis patients and HLA DRB1 allele polymorphisms. We hypothesized that anti-CagA IgA would be increased for specific HLA-DRB1 types (indicating protection) and decreased for other specific HLA-DRB1 types (indicating susceptibility). To test this hypothesis, we analyzed the frequency of HLA-DRB1 alleles in a cross-sectional study in a Turkish patient cohort with gastritis symptoms who had available blood samples and histopathological tissue slides. After quantitating serum anti-CagA IgA and performing histopathological evaluations and gastroendoscopy (consistent with standard care), we observed informative correlations between DRB1 genotype and anti-CagA IgA positivity. For HLA-DRB1 genotypes of interest, we then utilized established in silico prediction methods to characterize their relative capacity to present CagA peptides to T cells.

## 2. Materials and Methods

### 2.1. Designing Gastritis Patient Recruitment Protocol

Patients who applied to Medipol University Esenler Hospital Gastroenterology Clinic for gastroendoscopy procedures were included as study group between March 2016 and February 2017 for 12-month period.

Approval was received from Istanbul Medipol University Non-invasive Clinical Research Ethics Committee with document numbers “10840098-604.01.01-E.28917” and “E-10840098-772.02-2621” for the collection of biological samples and demographic data of the patients. Patients’ diagnoses, treatment procedures, or standard of care were not interfered during data and sample collection. All patients underwent a gastroendoscopic procedure under general anesthesia and whole blood and serum samples were taken to examine the complete blood count (CBC) and hepatic markers for control purposes before this procedure. As biological samples were collected as part of routine diagnostic procedures, no additional samples were taken.

### 2.2. DNA Preparation and HLA Typing

By using the QIAamp whole-blood DNA mini kit and Isolation System (Qiagen, Hilden, Germany), genomic DNA was extracted from peripheral blood samples taken into sample tubes containing ethylenediaminetetraacetic acid (EDTA). In order to determine genomic DNA concentration and purity, the ultraviolet spectrophotometer measurements were used. DNA samples with 20–50 ng/μL DNA concentration, and 1.6–1.9 purity ratio at A260/280 nm were stored at +4 °C and analyzed in a week; otherwise, they were stored at −20 °C. For HLA typing, PCR (polymerase chain reaction SSO method; sequence-specific oligonucleotide probes, Luminex Technology, high-resolution typing) was performed. Sequence-specific oligonucleotide probes (PCR-SSO, Luminex; Tepnel Lifecodes, Stanford, CA, USA) were used for polymerase chain reaction (PCR) amplification. Extracted DNA samples with ≥10 ng DNA concentration were analyzed. HLA-DR e-res kits (Immucor Medicine Diagnostic GmbH, Dreieich Germany) were prepared for amplification. Master Mix (mixture containing Primer) (3.5 μL) distilled water (12.5 μL) and Taq polymerase (0.25 μL) were prepared for each HLA type and poured into 8 PCR tubes (Thermo Fisher Scientific™, Waltham, MA, USA) and DNA (5 μL) was added. The reaction was started by placing the 8 tubes prepared for the PCR cycle into the thermal cycler. PCR amplification was performed with an Applied Biosystems ProFlex Thermal Cycler (Thermo Fisher Scientific, Waltham, MA, USA) using the following PCR cycles. Then, 1 cycle for 3 min at 95 °C was followed by 12 cycles of 15 s at 95 °C, 30 s at 60 °C, and 30 s at 72 °C; followed by 28 cycles of 10 s at 95 °C, 30 s at 63 °C, and 30 s at 72 °C; next, the reaction continued for 2 min at 72 °C. Finally, fixed at +4.0 °C. After the amplification process, the samples were stored at temperatures ranging from +2 °C to +8 °C for 24 h until the end of the analysis period. Five microliters of amplified products was added to Costar plates. Beads in HLA-DR eRes kits were heated at 55 °C for 7 min using a sonication device (Ultrasonic cleaner, Branson 200, Branson Soest, Utrecht, The Netherlands) and then left at room temperature for 15 s. The beads were then vortexed (V-1 plus, Personal Vortex, Biosan, Riga, Latvia), and 15 μL of these beads and 5 μL of amplified product were transferred to the Costar plate and placed in the Applied Biosystems ProFlex Thermal Cycler (Thermo Fisher Scientific, Waltham, MA, USA). The dilution solution mixture was prepared near the end of the hybridization (including kits and streptavidin prepared for each costar plate), and 170 μL of this mixture was pipetted into to each well after the finalization of the hybridization process. The Costar plate was then loaded onto the Luminex device (Tepnel Lifecodes, Stanford, CA, USA), and the plate was automatically read in the system. Reading was recorded in the system and stored as raw data in the database. All the generated raw data were transferred to the analysis program (Match-IT ver 3.35), and HLA-DRB1 high-resolution results were obtained and all of them were recorded.

### 2.3. Measurement Method of Serum Anti-CagA IgA

Serum samples were collected and frozen at −50 °C until ELISA analysis. Serum concentrations of anti-CagA IgA (human IgA raised against the CagA protein of *H. pylori*) were analyzed by ELISA method. ELISA steps were followed according to the manufacturer’s protocol written in the kit insert (DiaPro Diagnostic Bioprobes Srl No: 27 20099, Milan, Italy).

### 2.4. Histopathological Evaluation of Metaplasia in Gastric Mucosa Biopsy Samples

During gastroendoscopy procedure, mucosal tissue biopsy samples were taken from the stomach. Biopsy samples were first fixed in 10% formalin and then cut into 4–6 mm pieces. After staining with Giemsa stain and hematoxylin and eosin, the presence of *H. pylori* was classified as positive or negative during the microscopic examination of tissue slides. Histopathological examination was performed by the same pathologist who was blinded to clinical and laboratory data.

### 2.5. Gastroendoscopy Procedure

In order to diagnose gastritis, all gastroendoscopy procedures were performed by a single gastroenterologist who was blinded to other study data. According to gastric mucosal appearance (viewed through a camera), gastric mucosa was classified as having normal mucosa, erythematous gastritis, erosive gastritis, or pangastritis with or without ulcer. Biopsy samples were collected according to the Sydney Protocol. Biopsy sites for the Sydney system are antrum (approximately 2 cm from the pylorus), incisura (center), lesser curve (mid-way between the incisura and cardia), and greater curve (central, opposite to lesser curve).

### 2.6. Prediction of CagA Peptide Presentation by DRB1 Alleles of Interest

We used two complementary approaches to compare the capacity of DRB1*03:01 and DRB1*11:04 to bind and present CagA peptides. First, we utilized a previously published prediction matrix method to scan the entire CagA protein sequence for minimal 9mer motifs with amino acid sequences likely to bind to DRB1*03:01 [28] or DRB1*11:04 [29]. Each matrix predicted a relative binding affinity (RBA) for all of the 1176 unique 9mer registers that exist within a CagA reference sequence (GenBank:ALF95202.1). Motifs with a predicted RBA < 0.1 were considered as non-binders and discarded. Remaining motifs were classified as nominal motifs (RBA > 0.1), moderate motifs (RBA > 0.33), or strong motifs (RBA > 0.99). Second, we accessed the immune epitopes database HLA class II binding prediction resource http://tools.iedb.org/mhcii/ (accessed on 16 February 2023) and utilized the IEDB recommended 2.22 prediction method [30] with default settings (15mer peptide size, peptides sorted by adjusted rank) to identify CagA peptides likely to bind to DRB1*03:01 or DRB1*11:04. Theoretical peptides were classified as having a nominal (Adj Rank < 50), moderate (Adj Rank < 10), or strong (Adj Rank < 3) likelihood of binding. These measures were collated to draw inferences about how effectively these two HLA molecules present CagA peptides to T cells. CagA peptides’ (15mers) sequence and binding affinity scores were also analyzed by using NetMHCIIPan 4.0 [31].

### 2.7. Statistical Analysis

SPSS (version 25) was used for statistical analysis. Student’s *t* test was used for comparisons between groups of normally distributed quantitative data; the Mann–Whitney U test was used for those that did not show a normal distribution. Differences in the allele frequencies of HLA-DRB1 between anti-CagA IgA-negative and IgA-positive groups were compared by chi-squared statistics (Pearson chi-square). Odds ratios (ORs) and 95% confidence intervals (95% CIs) were calculated for each allele. Correlations between parametric variables were determined by Spearman correlation. Logistic regression analysis was performed to determine the impact of DRB1 genotypes on anti-Cag A IgA positivity. Differences in the number of peptides predicted to be bound by the two HLA-DR alleles of interest were compared using a standard chi-squared test for approach 1 (too many observations for the Fisher exact test) and the Fisher exact test for approach 2.

## 3. Results

### 3.1. Demographic Characteristics, Histopathological Evaluation, and Gastroendoscopy Diagnosis of the Study Population

Patients who gave consent by filling out the consent form were included in the study (n = 292). Cases with comorbidities (n = 91) (diabetes mellitus, hypertension, hyperthyroidism, etc.), previous surgery history, cancer diagnosis and patients taking medication were excluded from the study. In addition, after excluding patients diagnosed with stomach ulcer and stomach cancer (n = 31), the study was conducted on patients diagnosed with erythematous gastritis, erosive gastritis, and pangastritis, as well as normal mucosa. Blood specimens (serum and whole blood in EDTA-containing tubes) were taken before the gastroendoscopic examination, and gastric tissue biopsies were obtained during gastroendoscopy (n = 170). Finally, in the current study, patients (n = 170) diagnosed with erytamotous and erosive gastritis without any comorbidities or continuous drug usage were enrolled.

The study consisted of 170 patients (53.5% (n = 91) female and 46.5% (n = 79) male) who were admitted to the Gastroenterology Clinic due to gastric complaints. The age of the cases ranged from 16 to 69 (37.2 ± 11.5 years). According to gastroendoscopy examination, 18 (10.6%) patients had normal gastric mucosa, and 152 patients were diagnosed with gastritis. Gastritis patients had subdiagnoses of erythematous gastritis (n = 121; 71.2%), erosive gastritis (n = 16; 9.4%), and pangastritis (n = 15; 8.8%). *H. pylori* was positive in 124 (72.9%) patients’ gastric mucosa tissues, whereas 46 tissue slides were negative (27.1%), histopathologically. Serum anti-CagA IgA positivity rate was in total 40/170 (23.2%). Among 46 *H. pylori*-negative patients, anti-CagA IgA positivity rate was 13.0% (6 out of 46), and among 124 *H. pylori*-positive patients, anti-CagA positivity rate is 27.4% (34 out of 124). The distribution of anti-CagA IgA among *H. pylori*-positive and -negative groups was not statistically significant (*p* = 0.066; Pearson chi-square test) (Table 1).

### 3.2. Allele Frequency of the Study Population and Allele Distribution among the Anti-CagA IgA-Positive and H. pylori-Positive Groups

HLA typing detected a total of 40 different HLA-DRB1 types in our study group, with percentages as listed in Table 2. The detection rate of 17/40 was greater than 2%. In the genetic background of the group we studied, people defined themselves as Turkish. The most common DRB1 allele by percentage was found as *11:04 (15% of the study population). Additional common alleles (more than 2 percent of the study population) were *11:01 (10.6%), *03:01 (9.1%), *07:01 (8.5%), *13:01 (5.6%), *15:01 (5.3%), *04:03 (4.1%), *14:01 (3.8%), *15:02 (3.8%), *16:01 (3.2%), *10:01 (2.9%), *01:01 (2.4%), *01:02 (2.4%), *04:03 (2.4%), *04:02 (2.4%), *14:54 (2.4%), *08:03 (2.1%), *13:02 (2.1%). Anti-CagA IgA serum was positive in 40 (23.6%) out of 170 patients. The resulting allele pool is 340 (170 patients × 2). As indicated in Table 2, individuals with the HLA-DRB1 *03:01 allele (2n = 31; 9.1%) were found to be significantly more likely to have positive anti-CagA IgA: 17 negatives (6.54%) vs. 14 positives (17.50%) (Pearson chi-square value: 8.871; *p* = 0.030). No other allele showed a significant association with anti-CagA IgA. However, it should be noted that there was a trend toward positive anti-CagA IgA antibody in individuals carrying the DRB1 *07:01 allele (Pearson chi-square value = 3.655; *p* = 0.056). Individuals carrying the DRB1*11:04 allele were less likely to have the positive anti-CagA IgA antibody (Pearson chi-square value = 3.206; *p* = 0.073) (Table 2).

In 124 out of the 170 patients (72.9%), *H. pylori* was found to be positive by the histopathological analysis of gastric mucosal biopsies. When the polymorphisms in the entire allele pool were examined, the presence of *H. pylori* was significantly associated with the presence of the HLA-DRB1*11:04 allele (n = 8; 8.70% vs. n = 43; 17.34%) (Pearson chi-square value = 3.932; *p* = 0.047) (Table 2). The histopathological presence of *H. pylori* in the gastric mucosa was not significantly associated with any other HLA-DRB1 polymorphisms.

### 3.3. Genotype Analysis of HLA-DRB1 *03:01 and *11:04 Polymorphisms with H. pylori and Anti-CagA IgA

Not surprisingly, among the 31 (out of 170) patients who were HLA-DRB1*03:01 positive, some individuals were heterozygous whereas others were homozygous. We found that anti-CagA IgA production was significantly increased in individuals who were either heterozygous or homozygous for DRB1*03:01 (n = 14; 45.2%) compared to those who did not carry that allele (n = 26; 18.7%) (Pearson Chi Square value = 9.860; *p* = 0.002; OR = 3.579 (1.567–8.174) (Table 3). However, our data did not show any effect of DRB1*03:01 on the presence of *H. pylori* (n = 98; 70.5% vs. n = 26; 83.9%) (Pearson-chi square value = 2.294; *p* = 0.129; OR = 2.175 (0.781–6.058) (Table 3). Among the 47 (out of 170) patients who were HLA-DRB1*11:04-positive, some individuals had the heterozygous genotype whereas others had the homozygous genotype. We found that anti-CagA IgA production was significantly decreased in individuals who were either heterozygous or homozygous for DRB1*11:04 compared to those who were not (n = 34; 27.6% vs. n = 6; 12.8%) (Pearson chi-square value = 4.183; *p* = 0.041; OR = 0.383 (0.149–0.984) (Table 3). Interestingly, the incidence of *H. pylori* was significantly increased in individuals who were heterozygous or homozygous for the DRB1*11:04 polymorphism compared to those who were not (n = 40; 85.1% vs. n = 84; 68.3%) (Pearson chi-square value = 4.871; *p* = 0.027; OR = 2.653 (1.091–6.449) (Table 3).

Examining the antibody data in a quantitative fashion, in the presence of DRB1 *03:01 (heterozygous or homozygous carriers), the serum anti-CagA IgA increased 3.665-fold (*p* = 0.006; 95% CI 1.409–7.832). In the presence of DRB1*11:04 (heterozygous or homozygous carriers), the serum anti-CagA IgA was significantly reduced 0.373-fold (*p* = 0.024; 95% CI 0.121–0.861). In the presence of HLA-DRB1*11:04 (heterozygous or homozygous carriers), *H. pylori* positivity in the gastric mucosa was significantly increased 2.717-fold (*p* = 0.028, 95% CI 1.112–6.638; logistic backward odds ratio analysis). In the presence of HLA DRB1 *03:01 (heterozygous or homozygous carriers), there was no significant effect on *H. pylori* positivity in the gastric mucosa (*p* = 0.122; logistic regression analysis-backward stepwise likelihood ratio) (Table 4).

### 3.4. Predicting the Relative Capacity of HLA-DRB1*03:01 and HLA-DRB1*11:04 to Present CagA Peptides

The observed associations between anti-CagA IgA formation and two HLA-DRB1 haplotypes of interest (positive for DRB1*03:01 and negative for DRB1*11:04) and the concurrent observation that the incidence of *H. pylori* is increased in DRB1*11:04 positive individuals suggest that the effective presentation of CagA-derived peptides to T cells may be crucial for IgA formation. As detailed in the methods section, we utilized two complementary approaches to test the hypothesis that HLA-DRB1*03:01 and HLA DRB1*11:04 differ in their ability to present CagA peptides. Although not all differences were significant, the two methods converged to suggest that DRB1*03:01 has a greater capacity to present CagA peptides to T cells. The first method scanned all possible 9mer sequences (1176 total) to define peptides with nominal, moderate, or strong motifs consistent with the known binding preferences of the two HLA-DR alleles of interest. In this analysis, a higher predicted RBA indicates better binding. Consistent with our expectation, this analysis predicted higher numbers of peptides for DRB1*03:01 than DRB1*11:04 with nominal (191 versus 158, *p* = 0.056), moderate (109 versus 78, *p* = 0.018), or strong (32 versus 19, *p* = 0.066) motifs. The second computed the likelihood of binding for all possible 15mer peptides (737 total). In this analysis, a lower adjusted rank indicates better binding. Consistent with our expectation, this analysis predicted higher numbers of peptides for DRB1*03:01 than DRB1*11:04 with nominal (434 versus 416, *p* = 0.34), moderate (70 versus 58, *p* = 0.27), or strong (13 versus 0, *p* = 0.0013) adjusted binding ranks (Table 5).

Amino acid sequences of 15-peptide CagA fragments which have the highest capacity (<2% rank eluted ligand (EL) prediction score) of HLA-DRB1*11:04 and DRB1*03:01 polymorphisms were evaluated by using NetMHCIIPan 4.0 (Table 6). DRB1*11:04 allele carriers who had a significantly lower anti-CagA IgA and *H. pylori* positivity could bind 3 of 15mer peptides of CagA, whereas DRB1*03:01 carriers who had significantly higher serum anti-CagA IgA could bind 15 of 15mer peptides in <1% Rank EL. When the sequence of 15mer peptides of CagA which could bind to DRB1*11:04 and *03:01 within <2% Rank EL were listed, only VIINQKITD (position of amino acid residue number = 775) core peptide was common to both of these two alleles (Table 6).

## 4. Discussion

*H. pylori* is associated with gastric inflammation and gastric cancer [32,33]. The reasons that *H. pylori* infection can elicit such diverse outcomes in different individuals remain elusive; CagA expression has been linked to increased pathogenicity [16] but human and murine studies suggest a role for antibodies and T cell immunity in controlling infection and inflammation [6,19,20]. In this study, we asked whether specific HLA-DR genotypes are associated with, *H. pylori* infection, and anti-CagA seropositivity. Given that each HLA protein binds and presents a distinct array of peptides to T cells, we hypothesized that distinct alleles would be associated with the increased or decreased immune recognition of *H. pylori* CagA protein. Consistent with that expectation, we observed opposing tendencies for gastritis patients carrying HLA-DRB1*03:01 versus HLA-DRB1*11:04 genotypes. There was a significant positive association between DRB1 *03:01 and positive anti-CagA IgA, implicating this as a protective genotype. In contrast, the presence of *H. pylori* in the gastric mucosa was significantly increased in individuals carrying the *11:04 genotype and DRB1*11:04 was negatively associated with anti-CagA IgA. These clinical data prompted us to examine the capacity of DRB1*11:04 and DRB1*03:01 to present CagA peptides to T cells. Notably, two complementary in silico prediction methods agreed that DRB1*03:01 is expected to present more CagA peptides than DRB1*11:04. Although additional experiments would be required to define immunogenic CagA peptides that elicit CD4+ T cell responses, these findings support a paradigm in which more effective T cell help in individuals who are DRB1*03:01 positive (as compared to DRB1*11:04 positive individuals) leads to the effective production of anti-CagA IgA to oppose *H. pylori* infection and its associated inflammation.

In our study, we observed positive *H. pylori* infection in 72.9% of our patient cohort, but only 27.4% had a positive anti-CagA IgA antibody (Table 1). In agreement with our data, a study by Yılmaz et al. (2006) [34] observed total anti-CagA IgA positivity in 17/56 (30%) of their Turkish cohort, including anti-CagA IgA positivity in 16/48 (33%) of their *H. pylori*-positive group. Some prior studies have concluded that serological findings lacked value for diagnosing *H. Pylori* infection [35,36]. However, the presence of anti-CagA has been associated with clinical outcome [37]. The topic of HLA associations and *H. Pylori* infection remains controversial. It is a generally held view that human genetic variants such as HLA class II are useful predictors of antibody responses to common pathogens and vaccines [38]. A meta-analysis of *H pylori* infection in Asian and European populations identified protective and susceptible HLA-DQ genotypes in Asian populations only [39]. Other studies found no significant correlations between HLA class II genotype, CagA-specific antibodies, or *H pylori* infection [37,40]. The first published study in a Turkish population observed univariate correlations between HLA-DQA1*01 susceptibility to gastric cancer and between HLA-DQA1*05 and protection [41]. Their multivariate modeling revealed HLA-DQA1*01 and HLA-DQB1*06 as risk haplotypes and HLA-DRB1*04 as a protective haplotype [41]. However, it should be noted that the size of their cohort was modest (94 patients and 86 controls) given the complexity of their analysis. The associations we observed were distinct from those seen in previous studies, but harmonize with the general concept that specific HLA class II haplotypes could be protective or increase susceptibility to specific pathogens.

Specific HLA-DR genotypes are also known to be strongly associated with several autoimmune conditions [42] and with susceptibility and resistance to infectious diseases [43]. Specific HLA-DR alleles are closely linked to HLA-DQ alleles and this haplotype linkage often makes it difficult to resolve the causative factor that is associated with disease. Pertaining to our findings, the DR-11 serotype is linked with the DQ7 serotype. HLA DRB1*11:04 was co-inherited with HLA DQA1*05:05-DQB1*03:01 haplotype in 2.5% frequency among Americans of European descent [44]. The DRB1*11:04 allele was found to be significantly correlated with both DQA1*05:05 and DQB1*03:01 alleles as well as with the DQA1*05:05~DQB1*03:01 haplotype in our study population (unpublished data) [45]. However, since these patients are diagnosed as having gastritis, all these alleles might be enriched in our cohort. Thus, regardless of the underlying patterns of inheritance, DR is a distinct protein which can selectively bind CagA peptide fragments. To verify clinical data which suggest that carrying the HLA-DR*11:04 allele might cause lower anti-CagA IgA production, in silico methods were performed. According to the NetMHCIIPan 4.0 analysis [31], it was predicted that the HLA-DRB1*11:04 and DRB1*03:01 alleles might bind distinct sequences and different overall numbers of 15mer CagA peptides. When the anti-CagA IgA seropositivity is evaluated and an immunogen-based vaccine against *H. pylori* CagA is considered, individuals carrying the DRB1*11:04 allele might have less binding potential compared to DRB1*03:01 allele carriers.

Among 46 *H. pylori*-negative patients, the anti-CagA IgA positivity rate was 13.0% (6/46) (Table 1). The false negative sampling of the biopsy specimens of the gastric mucosa might be the reason for the discrepancy. In addition, *H. pylori* might also reside in other intestinal regions and might affect anti-CagA IgA positivity in serum.

We acknowledge that our study had limitations. For example, we histopathologically evaluated the presence of *H. pylori* in gastric mucosa sections, but we were not able to evaluate whether *H. pylori* from the sections were CagA-positive. Consequently, we do not know the percentage of our cohort that was infected with CagA positive *H. pylori*. One further complication is that, as reported by Fakhre-Yaseri et al. (2016) [46], only 62.8% of dyspeptic patients infected with the CagA positive strain produced serum anti-CagA IgA and CagA positive *H. pylori* infection and serum anti-CagA IgA were not well correlated, making this antibody a weak tool for diagnosis [34]. Another key weakness is our inability to perform detailed T cell studies to further conform our findings. This omission was due to practical limitations (for example, the early stages of epitope discovery require fairly large blood volumes). However, we consider such efforts to be an important direction for future research efforts.

## 5. Conclusions

In this cross-sectional study of a Turkish population of gastritis patients, significantly less anti-CagA IgA in serum was present in HLA-DR*11:04 positive gastritis cases. In cases carrying the HLA-DR*03:01 genotype, significantly more serum anti-CagA IgA was detected. Correspondingly, HLA-DRB1*03:01 was predicted to have a much greater capacity than DRB1*11:04 to present CagA peptides to CD4+ T cells. In combination, our clinical findings about anti-CagA IgA and predicted differences in CagA peptide presentation suggest that these are distinct susceptible and protective HLA-DR genotypes in our population.

## Figures and Tables

**Table 1:** Details of demographic characteristics, histopathological examination findings, gastroendoscopy diagnosis, serum anti-CagA IgA presence among the study group.

Parameters		Study Group (n = 170)
Age (years)	Mean ± S.D.	37.2 ± 11.5
	Median (min–max)	36.0 (16–69)
Sex	Female	91 (53.5%)
	Male	79 (46.5%)
Stomach endoscopy diagnosis	Normal mucosa	18 (10.6%)
	Gastritis subgroups	
	-erythematous	121 (71.2%)
	-erosive	16 (9.4%)
	-pangastritis	15 (8.8%)
*H. pylori*, histopathology	Negative	46 (27.1%) (anti-CagA IgA positive 6/46; 13.0%)
	Positive	124 (72.9%) (anti-CagA IgA positive 34/124; 27.4%)
anti-CagA IgA, serum	Negative	130 (76.8%)
	Positive	40 (23.2%)

**Table 2:** The total allele distribution of HLA-DRB1 among the study group, and patients with anti-CagA IgA-negative and -positive groups as well as *H. pylori*-negative and -positive groups.

HLA DRB1 Alleles	Total Allele Frequency (%) 2N = 340	Anti-Cag A IgA Negative 2N = 260 (76.4%) AF (%)	Anti-Cag A IgA Positive 2N = 80 (23.6%) AF (%)	*H. pylori* Negative 2N = 92 (27.1%) AF (%)	*H. pylori* Positive 2N = 248 (72.9%) AF (%)
*01:01	8 (2.4)	5 (1.92)	3 (3.75)	2 (2.17)	6 (2.42)
*01:02	8 (2.4)	6(2.31)	2 (2.50)	2 (2.17)	6 (2.42)
*03:01	31 (9.1)	17(6.54)	14 (17.50)	5 (5.43)	26 (10.48)
Pearson χ^2^ *p*		^b^ 8.871 0.030		n.s.	
*03:06	1 (0.3)	1 (0.38)	0 (0)	0 (0)	1 (0.40)
*04:01	6 (1.8)	4 (1.54)	2 (2.50)	0 (0.00)	6 (2.42)
*04:02	8 (2.4)	6 (2.31)	2 (2.50)	4 (4.35)	4 (1.61)
*04:03	14 (4.1)	9 (3.46)	5 (6.25)	5 (5.43)	9 (3.63)
*04:04	5 (1.5)	5 (1.92)	0 (0.00)	3 (3.26)	2 (0.81)
*04:05	1 (0.3)	0 (0.00)	1 (1.25)	0 (0.00)	1 (0.40)
*04:07	3 (0.9)	3 (1.15)	0 (0.00)	2 (2.17)	1 (0.40)
*04:08	2 (0.6)	1 (0.38)	1 (1.25)	1 (1.09)	1 (0.40)
*04:13	1 (0.3)	1 (0.38)	0 (0.00)	0 (0.00)	1 (0.40)
*07:01	29 (8.5)	18 (6.90)	11 (13.75)	11 (11.96)	18 (7.26)
Pearson χ^2^ *p*		^b^ 3.655 0.056		n.s.	
*08:03	7 (2.1)	5 (1.92)	2 (2.50)	2 (2.17)	5 (2.02)
*09:01	1 (0.3)	1 (0.38)	0 (0.00)	0 (0.00)	1 (0.40)
*10:01	10 (2.9)	7 (2.69)	3 (3.75)	2 (2.17)	8 (3.23)
*10:22	1 (0.3)	1 (0.38)	0 (0.00)	0 (0.00)	1 (0.40)
*11:01	36 (10.6)	29 (11.15)	7 (8.75)	8 (8.70)	28 (11.29)
*11:03	3 (0.9)	1 (0.38)	2 (2.50)	1 (1.09)	2 (0.81)
*11:04	51 (15.0)	44 (16.92)	7 (8.75)	8 (8.70)	43 (17.34)
Pearson χ^2^ *p*		^b^ 3.206 0.073		^a^ 3.932 0.047	
*11:46	1 (0.3)	1 (0.38)	0 (0.00)	0 (0.00)	1 (0.40)
*11:62	3 (0.9)	3 (1.15)	0 (0.00)	1 (1.09)	2 (0.81)
*12:01	6 (1.8)	4 (1.54)	2 (2.50)	3 (3.26)	3 (1.21)
*12:14	1 (0.3)	1 (0.38)	0 (0.00)	1 (1.09)	0 (0.00)
*13:01	19 (5.6)	17 (6.54)	2 (2.50)	4 (4.35)	15 (6.05)
*13:02	7 (2.1)	6 (2.31)	1 (1.25)	4 (4.35)	3 (1.21)
*13:03	5 (1.5)	5 (1.92)	0 (0.00)	1 (1.09)	4 (1.61)
*13:05	1 (0.3)	1 (0.38)	0 (0.00)	0 (0.00)	1 (0.40)
*13:143	1 (0.3)	1 (0.38)	0 (0.00)	0 (0.00)	1 (0.40)
*14:01	13 (3.8)	11 (4.23)	2 (2.50)	4 (4.35)	9 (3.63)
*14:05	1 (0.3)	1 (0.38)	0 (0.00)	0 (0.00)	1 (0.40)
*14:54	8 (2.4)	6 (2.31)	2 (2.50)	3 (3.26)	5 (2.02)
*14:172	1 (0.3)	0 (0.00)	1 (1.25)	0 (0.00)	1 (0.40)
*15:01	18 (5.3)	16 (6.15)	2 (2.50)	9 (9.78)	9 (3.63)
*15:02	13 (3.8)	12 (4.62)	1 (1.25)	2 (2.17)	11 (4.44)
*15:03	1 (0.3)	1 (0.38)	0 (0.00)	1 (1.09)	0 (0.00)
*16:01	11 (3.2)	7 (2.69)	4 (5.00)	2 (2.17)	9 (3.63)
*16:02	2 (0.6)	2 (0.77)	0 (0.00)	1 (1.09)	1 (0.40)
*16:09	1 (0.3)	1 (0.38)	0 (0.00)	0 (0.00)	1 (0.40)
*16:25	1 (0.3)	0 (0.00)	1 (1.25)	0 (0.00)	1 (0.40)

^a^ *H. pylori*-negative group vs. -positive group comparison. ^b^ Anti-CagA IgA-negative group vs. -positive group comparison. AF: allele frequency. n.s. not significant.

**Table 3:** Distribution of DRB1*03:01 and DRB1*11:04 genotypes among *H. pylori* and anti-CagA IgA groups.

	*H. pylori*, Gastric Mucosa		Anti-CagA IgA, Serum	
HLA DRB1 Genotypes	Negative N = 46 (27.1%)	Positive N = 124 (72.9%)	Negative N = 130 (76.5%)	Positive N = 40 (23.5%)
DRB1*03:01−/DRB1*03:01−	41 (29.5%)	98 (70.5%)	113 (81.3%)	26 (18.7%)
DRB1*03:01+/DRB1*03:01−+	5 (16.1%)	26 (83.9%)	17 (54.8%)	14 (45.2%)
Pearson χ^2^ 2-tailed *p* Odds ratio (OR)	^a^ 2.294 0.129 2.175 (0.781–6.058)		^b^ 9.860 0.002 3.579 (1.567–8.174)	
DRB1*11:04−/DRB1*11:04−	39 (31.7%)	84 (68.3%)	89 (72.4%)	34 (27.6%)
DRB1*11:04+/DRB1*11:04−+	7 (14.9%)	40 (85.1%)	41 (87.2%)	6 (12.8%)
Pearson χ^2^ 2-tailed *p* Odds ratio (OR)	^a^ 4.871 0.027 2.653 (1.091–6.449)		^b^ 4.183 0.041 0.383 (0.149–0.984)	

^a^ *H. pylori*-negative group vs. -positive group comparison. ^b^ Anti-CagA IgA-negative group vs. -positive group comparison.

**Table 4:** Effect of HLA-DRB1*03:11 and *11:04 allele carriers on *H. pylori* and serum anti-CagA IgA.

Dependent: Anti-CagA IgA, Serum		B	S.E.	Wald	df	Sig.	Exp(B)	95% C.I. for Exp(B)	
								Lower	Upper
Step 1	^a^ DRB1*03:01	1.200	0.438	7.524	1	0.006	3.322	1.409	7.832
	^b^ DRB1*11:04	−1.130	0.500	5.108	1	0.024	0.323	0.121	0.861
	*H. pylori*	0.984	0.499	3.883	1	0.049	2.675	1.005	7.120
	Constant	−1.958	0.455	18.493	1	0.000	0.141		
**Dependent:** ***H. pylori*, Gastric Mucosa**		**B**	**S.E.**	**Wald**	**df**	**Sig.**	**Exp(B)**	**95% C.I. for Exp(B)**	
								**Lower**	**Upper**
Step 1 ^a^	^a^ DRB1*03:01	0.816	0.528	2.389	1	0.122	2.262	0.804	6.369
	^b^ DRB1*11:04	0.999	0.456	4.808	1	0.028	2.717	1.112	6.638
	Constant	0.635	0.208	9.317	1	0.002	1.887		

Method: logistic regression analysis, backward stepwise method (likelihood ratio). ^a^ DRB1* 03:01 Genotype was coded as negatives for both alleles vs. positive for at least one allele or both alleles. ^b^ DRB1* 11:04 Genotype was coded as negatives for both alleles vs. positive for at least one allele or both alleles.

**Table 5:** Predicted binding of CagA peptides by HLA-DR alleles of interest.

HLA Allele	Nominal Motifs (RBA > 0.1)	Moderate Motifs (RBA > 0.33)	Strong Motifs (RBA > 0.99)	IEDB Nominal (Adj Rank < 50)	IEDB Moderate (Adj Rank < 10)	IEDB Strong (Adj Rank < 3)
DRB1*03:01	191	109	32	434	70	13
DRB1*11:04	158	78	19	416	58	0
*p*-value	0.056	0.018	0.066	0.37	0.30	0.0002

**Table 6:** The 15mers of CagA protein which could bind HLA-DRB1*11:04 and DRB1*03:01 with the <2% rank EL binding affinity by using NetMHCIIPan 4.0 prediction method.

HLA-DRB1 Alleles	Clinical Feature	Pos ^§^	15mers of CagA (Total Number = 1170)	Peptide Core	Score EL	% Rank EL
*11:04	-Anti-CagA IgA, in serum decreased -*H. pylori*, in gastric mucosa, increased	382	EDQLIGLKQALSQKE	LIGLKQALS	0.844626	0.65
		103	GSSIKSFQKFGTQRY	IKSFQKFGT	0.844022	0.65
		102	TGSSIKSFQKFGTQR	IKSFQKFGT	0.830930	0.72
		381	KEDQLIGLKQALSQK	LIGLKQALS	0.781132	1.01
		444	GNDHIAFVSKKDKKH	IAFVSKKDK	0.781062	1.01
		775	IKDVIINQKITDKVD	VIINQKITD	0.777171	1.03
		656	KDKIFAIINEEAGKE	IFAIINEEA	0.775886	1.04
		688	SDKLENINKNLKDFD	LENINKNLK	0.756505	1.15
		445	NDHIAFVSKKDKKHL	IAFVSKKDK	0.734347	1.31
		1003	KQKIDNLNQAVSEAK	IDNLNQAVS	0.723402	1.38
		1066	HIRINSNVKNGAINE	INSNVKNGA	0.709862	1.50
		774	SIKDVIINQKITDKV	VIINQKITD	0.694615	1.59
		893	NEPIYAKVNKKKTGE	YAKVNKKKT	0.688278	1.63
		655	QKDKIFAIINEEAGK	IFAIINEEA	0.686480	1.65
		687	LSDKLENINKNLKDF	LENINKNLK	0.673650	1.74
		467	NGELSYTLKDYGKKQ	LSYTLKDYG	0.673148	1.74
		657	DKIFAIINEEAGKEA	FAIINEEAG	0.666546	1.80
		1065	SHIRINSNVKNGAIN	INSNVKNGA	0.657271	1.88
		894	EPIYAKVNKKKTGEV	YAKVNKKKT	0.652250	1.92
		101	DTGSSIKSFQKFGTQ	IKSFQKFGT	0.650966	1.93
		443	LGNDHIAFVSKKDKK	IAFVSKKDK	0.648912	1.94
		776	KDVIINQKITDKVDN	VIINQKITD	0.642663	1.99
*03:01	-Anti-CagA IgA, in serum increased -*H. pylori*, in gastric mucosa, not changed	173	GNQIQSDQKFMGVFD	IQSDQKFMG	0.976363	0.02
		172	IGNQIQSDQKFMGVF	IQSDQKFMG	0.968244	0.03
		171	IIGNQIQSDQKFMGV	IQSDQKFMG	0.962267	0.05
		174	NQIQSDQKFMGVFDE	IQSDQKFMG	0.941053	0.11
		27	VAFLKVDNAVASFDP	LKVDNAVAS	0.872526	0.35
		170	IIIGNQIQSDQKFMG	IQSDQKFMG	0.862490	0.38
		428	EIEDFQKDSKAYLDA	FQKDSKAYL	0.840500	0.44
		26	QVAFLKVDNAVASFD	LKVDNAVAS	0.819071	0.50
		429	IEDFQKDSKAYLDAL	FQKDSKAYL	0.816953	0.51
		36	VASFDPDQKPIVDKN	FDPDQKPIV	0.795296	0.60
		427	NEIEDFQKDSKAYLD	FQKDSKAYL	0.777056	0.67
		35	AVASFDPDQKPIVDK	FDPDQKPIV	0.763118	0.72
		28	AFLKVDNAVASFDPD	LKVDNAVAS	0.740305	0.82
		430	EDFQKDSKAYLDALG	FQKDSKAYL	0.701666	0.98
		175	QIQSDQKFMGVFDES	IQSDQKFMG	0.697817	0.99
		37	ASFDPDQKPIVDKND	FDPDQKPIV	0.673853	1.11
		25	LQVAFLKVDNAVASF	LKVDNAVAS	0.662941	1.16
		34	NAVASFDPDQKPIVD	FDPDQKPIV	0.645142	1.24
		1093	WLKLVNDKIVAHNVG	LVNDKIVAH	0.606726	1.43
		775	IKDVIINQKITDKVD	VIINQKITD	0.551265	1.73
		494	QGNLKHDGVMFVNYS	LKHDGVMFV	0.540771	1.78
		1092	EWLKLVNDKIVAHNV	LVNDKIVAH	0.528630	1.85
		553	TSYIRRDLEDKLWAK	IRRDLEDKL	0.514902	1.94

% Rank EL: percentile rank of eluted ligand prediction score. Score EL: eluted ligand prediction score. ^§^ Pos: Position of residue number (starting from 0).

## Data Availability

The histopathological data generated analyzed during the current study are not publicly available because these represent confidential clinical data. All other data generated or analyzed during this study are included in this published article.
